# Supplementary material for: Non-Pharmacological Interventions on Pain in Amyotrophic Lateral Sclerosis Patients: A Systematic Review and Meta-Analysis
Source: Healthcare (Basel). 2024 Apr 1;12(7):770. doi: 10.3390/healthcare12070770 (PMC11011838; doi:10.3390/healthcare12070770)
Supplement: Supplementary file 1 [file healthcare-12-00770-s001.zip › healthcare-2903315-supplementary.pdf]

SUPPLEMENTARY MATERIAL

Table S1. Search Equation.

DATABASE	SEARCH QUERY	RESULTS
PUBMED	(((((ALS) AND (AMYOTROPHIC LATERAL SCLEROSIS)) AND (MOTOR NEURON DISEASE)) AND (LOU GEHRIG)) OR (PAIN)) AND (SMALL FIBER NEUROPATHY))))	858
SCOPUS	als AND amyotrophic AND lateral AND sclerosis AND motor AND neuron AND disease AND lou AND gehrig OR pain OR small OR fiber OR neuropathy	131
WEB OF SCIENCE	"ALS) AND AMYOTROPHIC LATERAL SCLEROSIS AND MOTOR NEURON DISEASE AND LOU GEHRIG OR PAIN AND SMALL FIBER NEUROPATHY"	4
COCHRANE	"ALS AND AMYOTROPHIC LATERAL SCLEROSIS AND MOTOR NEURON DISEASE AND LOU GEHRIG OR PAIN AND SMALL FIBER NEUROPATHY"	0
